# Supplementary material for: DNA methylation and gene expression changes derived from assisted reproductive technologies can be decreased by reproductive fluids
Source: eLife. 2017 Feb 1;6:e23670. doi: 10.7554/eLife.23670 (PMC5340525; doi:10.7554/eLife.23670)
Supplement: Supplementary file 1. — DOI: http://dx.doi.org/10.7554/eLife.23670.019 [file elife-23670-supp1.docx]

**Table S1.** Top Canonical Pathways, Physiological Systems and Molecular and Cellular Functions related to DEGs between blastocysts produced *in vitro* under two different systems.

| Top pathways |  |  |  |  | |
| --- | --- | --- | --- | --- | --- |
|  | **623 DEG (Natur-IVF)** | | **787 DEG (C-IVF)** | | |
| Upregulated genes | -log(B-H p-value) | Overlap | -log(B-H p-value) | Overlap | |
| Superpathway of Cholesterol Biosynthesis | 5,18 | 8/28 | 8,49 | 11/28 | |
| Cholesterol Biosynthesis I | 2,45 | 4/13 | 4,77 | 6/13 | |
| Superpathway of Geranylgeranyldiphosphate Biosynthesis I (via Mevalonate) | 2,22 | 4/17 | 2,68 | 5/17 | |
| p53 Signaling | 2,45 | 9/98 | 2,39 | 10/98 | |
| Serine Biosynthesis | 2,45 | 3/5 | 2,22 | 3/5 | |
| Mevalonate Pathway I | 1,46 | 3/13 | 2,13 | | 4/13 |
| Superpathway of Serine and Glycine Biosynthesis | 2,22 | 3/-- | 1,79 | | 3/-- |
|  |  |  |  |  | |
| Down regulated genes |  |  |  |  | |
| 14-3-3-mediated Signaling | -- | 0 | 1,4 | 7/117 | |
| Protein Ubiquitination Pathway | -- | 0 | 1,4 | 10/255 | |

Cuttof -log(B-H p-value) > 1.3

| Top Cellular functions | 623 (Natur-IVF) | | 787 (C-IVF) |
| --- | --- | --- | --- |
| Downregulated genes | No. molecules | | No molecules |
|  |  | |  |
| RNA Post-Transcriptional Modification | 24 | | 24 |
| DNA Replication, Recombination, and Repair | 30 | | 39 |
| Cell Morphology | 18 | | 13 |
| Cellular Function and Maintenance | 19 | | 27 |
| Cellular Compromise | 16 | | 15 |
| Cellular Growth and Proliferation | 69 | | 89 |
| Gene Expression | 46 | | 66 |
| Cell Cycle | 35 | | 52 |
| Cell Death and Survival | 58 | | 79 |
| Cellular Development | 48 | | 58 |
| Upregulated genes |  | |  |
| Cell movement | 108 | | 130 |
| Cell Death and Survival | 139 | | 175 |
| Cellular Growth and Proliferation | 153 | | 199 |
| Lipid Metabolism | 85 | | 107 |
| Molecular Transport | 95 | | 134 |
| Small Molecule Biochemistry | 94 | | 128 |
| Cell cycle | | 55 | 47 |
| Cellular Assembly and organization | | 82 | 107 |
| Cellular morphology | | 121 | 149 |
| Post-translational modification | | 38 | 43 |

| Top “Disease and Disorders” and “Physiological System Development and Function” | 623 (Natur-IVF) | 787 (C-IVF) |
| --- | --- | --- |
| Downregulated genes | No. molecules | No. molecules |
| Cancer | 128 | 170 |
| Organismal Injury and Abnormalities | 132 | 175 |
| Hereditary Disorder | 39 | 41 |
| Organ Morphology | 20 | 13 |
| Reproductive System Development and Function | 18 | -- |
|  |  |  |
| Upregulated genes |  |  |
| Tissue Development | 95 | 153 |
| Immunological Disease | 37 | 118 |
| Developmental Disorder | 54 | 73 |
| Hereditary Disorder | 45 | 86 |
| Metabolic Disease | 77 | 94 |
| Neurological Disease | 91 | 124 |
| Organismal Development | 102 | 177 |
| Cancer | 317 | 398 |
| Organismal Injury and Abnormalities | 319 | 403 |
