# Supplementary material for: DNA methylation and gene expression changes derived from assisted reproductive technologies can be decreased by reproductive fluids
Source: eLife. 2017 Feb 1;6:e23670. doi: 10.7554/eLife.23670 (PMC5340525; doi:10.7554/eLife.23670)
Supplement: Supplementary file 2. — DOI: http://dx.doi.org/10.7554/eLife.23670.020 [file elife-23670-supp2.docx]

**Supplementary file 2**. Functions associated with the down regulated genes in porcine blastocysts produced under conventional IVF conditions (C-IVF), compared to blastocyts produced using Natur-IVF system or collected *In vivo*.

| **Gene** | **Gene expression (RKPM)** | | | **FC (log)*** | **FC*** | **Knockout Phenotype** | **References** |
| --- | --- | --- | --- | --- | --- | --- | --- |
|  | **C-IVF** | **Natur-IVF** | **In Vivo** |  |  |  |  |
| KIT | -2,23 | 2,40 | 3,53 | 0,02 | -54,18 | Embryonic lethality  Perinatal lethality,  Abnormal primordial germ cells  Infertility, Anemia,  Slow postnatal weight gain,  Cardiovascular system, abnormal heart development, mortality/aging  Endocrine/exocrine, hematopoietic, homeostasis, immune, integument, pigmentation, reproductive  Digestive/alimentary system, nervous system | <http://www.informatics.jax.org/allele/summary?phenotype=&nomen=c-kit&chromosome=any&cm=&coordinate=&coordUnit=bp>; Bernex F, et al. 1996. Development; Rubin BP, et al., 2005. Cancer Research; Blume-Jensen P, et al 2000. Nat Genetics; Ro S et al. 2010 Gastroenterology  van Berlo JH, et al., 2014. Nature  Kissel H, et al., 2000. EMBO  Klein S, et al., 2013. Nat Commun. |
| MPP6 | -3,00 | 1,59 | 1,58 | 0,04 | -23,81 | No Knockout. Knock down of MPP6 leads to an accumulation of 3′ end extended 5.8S rRNAs which could cause defects in pre-rRNA processing into mature | Schilders G et al., 2005 Nucleic Acid Res  Yoshikatsu Y et al., 2015 Biochem Biophys Res Commun |
| MTA3 | -0,12 | 3,19 | 3,43 | 0,09 | -11,71 | Behavior, hematopoietic, homeostasis, immune | Skarnes WC et al., Nature 2011 |
| KIF4A | 4,20 | 6,39 | 7,19 | 0,13 | -7,94 | No abnormal phenotype observed in knockout. Functional knock down of KIF4A alters the rapid and correct attachment of chromosomes and cellular proliferation of KIF4A knockdown cells decreased significantly | Wandke et al., 2012. JCB  Minakawa Y et al., 2013. Plos One |
| UBR2 | 1,65 | 4,82 | 4,51 | 0,14 | -7,26 | Females died as embryos, whereas males were viable but infertile. Embryonic growth arrest, abnormal embryonic tissue physiology and morphology | Kwon YT, et al., Mol Cell Biol. 2003 Nov;23(22):8255-71  An JY et al., 2010. PNAS; An JY et al., 2012 Plos One |
| ISOC1 | 1,42 | 3,91 | 3,99 | 0,17 | -5,91 | Body weight loss | Phipps EL, Mp. Mouse News Lett. 1964;31:41;  Rainger J et al, 2013 Plos Genet; |

*FC (Fold Change) was calculated between Conventional IVF and In vivo gene expression data
