# Supplementary material for: DNA methylation and gene expression changes derived from assisted reproductive technologies can be decreased by reproductive fluids
Source: eLife. 2017 Feb 1;6:e23670. doi: 10.7554/eLife.23670 (PMC5340525; doi:10.7554/eLife.23670)
Supplement: Supplementary file 3. — DOI: http://dx.doi.org/10.7554/eLife.23670.021 [file elife-23670-supp3.docx]

**Supplementary file 3.** Percentages of specific features included in the 150 CpG size DMRs exclusive for each of three groups.

|  |  | **GROUPS** | | |
| --- | --- | --- | --- | --- |
| **Features** | **Global** | **C-IVF (*vs* Natur-IVF and *In-Vivo*)** | **Natur-IVF (*vs* C-IVF and *In-Vivo*)** | ***In-vivo* (*vs* Natur-IVF and C-IVF)** |
| CpG islands | 39961/258885  15.43% | 76/417  18.22% | 53/324  16.36% | 56/448  12.5% |
| Promoters | 31159/258885  12.03% | 30/417  7.19%  ** | 18/324  5.55%  ** | 32/448  7.14%  ** |
| TU | 108223/258885  41.8% | 185/417  44.36% | 153/324  47.22%  * | 185/448  41.29% |
| Intergenic | 180880/258885  69.87% | 267/417  64.03%  ** | 196/324  60.50%  ** | 295/448  65.85% |
| LINE1 | 162214/258885  62.66% | 236/417  56.59%  * | 195/324  60.18% | 271/448  60.49% |
| LTR | 119907/258885  46.32% | 169/417  40.53%  * | 128/324  39.5%  * | 231/448  51.56%  * |
| SINE | 143564/258885  55.45% | 214/417  51.32% | 170/324  52.47% | 231/448  51.56% |

* P-value <0.05

** P-value <0.01
