# Supplementary material for: DNA methylation and gene expression changes derived from assisted reproductive technologies can be decreased by reproductive fluids
Source: eLife. 2017 Feb 1;6:e23670. doi: 10.7554/eLife.23670 (PMC5340525; doi:10.7554/eLife.23670)
Supplement: Supplementary file 4. — DOI: http://dx.doi.org/10.7554/eLife.23670.022 [file elife-23670-supp4.docx]

**Supplementary file 4. T**op Molecular and Cellular Functions and representatives genes related to DMRs with higher or lower methylation in each group (C-IVF, Natur-IVF and *In vivo*).

|  | **DMRs with higher**  **methylation** | **DMRs with lower**  **methylation** |
| --- | --- | --- |
| **C-IVF** | **Death of embryo** (*PSMC3, KIF22, KIF5B*) | **Formation of embryonic tissue (***HTT*, L*RP8*, A*XIN1*, *PTCH1*, *TCOF1*, *FLT)* |
|  | **Apoptosis of embryonic cell lines** (*TNFRSF19, MAP3K1, AMOTL1*) | **Molecular mechanism of cancer (***AXIN1*, *JAK1*, *PTCH1*, *SYNGAP1, TFDP)* |
|  | **Apoptosis of embryonic cell lines** (*TNFRSF19, MAP3K1, AMOTL1*) | **Size of the embryo (***HTT*, *AXIN1*, *TFDP1*, *IGF2R*,*PTCH1*, *TCOF1* and *E2F8)* |
|  | **Formation of cellular protusions** (*CDC42BPB, ARAP1, MYO5A, VAV2, TRAF3IP1, KIF13B, MAP3K1, KIF5B, DSCAM, FIGF* and *RTN3*) | **STAT3/PTEN signaling (***IGF2R*, *LT1* and *TGFBR3)* |
|  |  | **Wnt/Ca+ pathway (***AXIN1* and *CREB1)* |
|  |  | **CNTFR signaling (***CNTFR* and *JAK1)* |
| **Natur-IVF** | *SUMO2, USP4, NR2C2 (TR4), TR2, TR4, ATF6, GSK3B, CSNK1A1, ABR, ANK3, ARHGEF11, CEP290, CRISPLD2, CTNNA2, IFT88, NDUFS4, NR2C2, RPSA* and*TLE1* | **Estrogen receptor signalling** (*CARM1, MED13, TAF4, TCF3, SUFU, POFUT2, CCNF, PRKG2,SHROOM3, PDGFRB* and *RUNX1*) |
| ***In vivo*** | **Zinc finger proteins** (*ZFP3, ZMYND8, ZNF200, ZNF250, ZNF608, ZNF609*) | **Solute carrier family members** (*SLC17A5, SLC22A8, SLC44A3, SLC4A2*) |
|  | *CAPRIN1*, *CD73*, *BOD1L*, *IRS1*, *MAP3K1*, E3, *PDGFA*, *PDGFD*, *STS*, *TCERG1* | *HSAFY*, *DCX* and *EIF4E* |
